# The Westminster Hospital Medical School

**Published:** 1910-12-31

**Authors:** 


### THE WESTMINSTER HOSPITAL MEDICAL SCHOOL.

The presentation of prizes at the above school took place in the board-room of the hospital on December 15, Lord Ilkeston handing the prizes to the recipients and subsequently giving an address. Sir John Wolfe-Barry, Chairman of the hospital, presided, and said that the Governors recognised to the full the immense advantages of a close alliance between the hospital and the school. The benefits of such an alliance were not appreciated as much as they should be by the world at large, but the advantage nevertheless was great and mutual. It was not possible to obtain a proper education for the medical student without the clinical teaching in the ward; the hospital gained by the increased efficiency which was always to be found when there was a system of teaching in connection with the institution, and the public gained most of all, because in a thorough medical education lay one of the great hopes of the future. The resources of the Westminster Hospital financially were not very great, and the Governors had been able to assist the school only in a moderate way, and in absolute accordance with the recommendation of Sir Edward Fry's committee—namely, that no money should be diverted from the sick and suffering poor to medical education unless with the consent of the subscribers. The school looked forward to further assistance from the alliance with the Board of Education, which was going to help the school in its finances and as a correlative thing take some control by representation on its committee.

The Dean (Mr. E. Rock Carling), in his report, said that there had been continued progress in every direction. The entries were better, and the work of the students, as evidenced in the examinations for which the prizes were awarded, and in the examinations of the licensing bodies, had been very good. More than thirty students had passed their qualifying examinations during the past two years. The arrangement with King's College whereby the earlier subjects of the curriculum were taught by the University continued to work successfully. The conditions which had embarrassed Metropolitan medical schools of late years were still in operation; but they confidently anticipated some measure of relief from the labours of the Royal Commission on University Education in London, which was now sitting. In the meantime the school had greatly improved its position and status by the recognition it had obtained from the Board of Education.

Lord Ilkeston then presented the prizes, which in many cases were of considerable monetary value. At the close of the distribution he said that he had been induced to come to the school that afternoon because during the past quarter of a century he had on many occasions visited Westminster Hospital to see sick colleagues of his, members of the House of Commons, who had been sent there by accident or illness, and he had greatly appreciated the kindness and promptitude of the medical staff, and because it gave him an opportunity of seeing again the keen, bright, eager faces of medical students. It was fifty years last October since he began to teach medical students, and for forty-two years, year in and year out, he was never without a class of students during the summer session at the Birmingham Medical School. During that time the profession and the students' curriculum had undergone many changes. Students now had enormous new tracts of learning to explore and to master, although on the other hand they had more splendid assistance in the shape of improved appliances and teaching. But if the teaching of students in the present day was more thorough and efficient, they had a harder and more difficult time when they started out in practice. It was true that there were larger opportunities than formerly in the public services; but, after all, general practice would be the professional destiny of most of them, and general practice was beset with more difficulties and greater competition than ever it had been before. It was not only in competition with public institutions, but it was threatened with a great State medical service, which was bound, if it materialised, to be a very grave competitor with the ordinary general practitioner.

"The general practitioner," said Lord Ilkeston, "is, after all, the backbone of our profession. He is the man I wish to guard and protect in his calling. I trust we may always have with us that noble class of men we have had in my lifetime—men who have done their work among all classes of the community with no hope of great pecuniary reward, but with perhaps the greatest of all rewards which comes from the knowledge that their lives were well-spent in alleviating the sufferings of others. The general practitioner in his daily work in the community is the representative of the medical profession. He is given the most responsible of all charges—the charge of our loved ones in times of suffering and danger. He enters the home as a friend and not as an official, and I should be sorry to see him replaced by any man acting as an officer of State, because the attention which can be given by a State official can never replace the personal devotion of the family doctor."

Some of the students, he continued, might doubt the attractiveness of general practice on the ground that it entailed a large amount of drudgery. But it was the drudgery in all callings which trained the best men. Lectures did not make practitioners, and it was the man who made a constant study of disease at close quarters, in the sick-ward and the out-patients' room, who found a most useful place in his profession.

In conclusion he said that if the connection between medical schools and hospitals were removed the public would be injured most of all. A hospital did the public good in two ways, first by relieving the applicant at its doors, and, secondly, by benefiting the mass of the public who did not come into direct touch with the institution. The rich man commanded the most skilled help of the physician, and, to put it on a low ground, he got better value for his money because the physician had been through a school which was attached to a hospital. The middle class also were benefited indirectly through the general raising of the whole tone of medical practice as a result of the combination of medical education with hospital work. In London, with its dozen medical schools, this aspect was apt to be overlooked, but in a provincial town the increased efficiency of the profession locally was very evident if a school were attached to the hospital, and the stimulus and inspiration which came from a medical staff employed in teaching were available.

